# Users of the main smartphone operating systems (iOS, Android) differ only little in personality

**DOI:** 10.1371/journal.pone.0176921

**Published:** 2017-05-03

**Authors:** Friedrich M. Götz, Stefan Stieger, Ulf-Dietrich Reips

**Affiliations:** 1 Department of Psychology, University of Konstanz, Konstanz, Germany; 2 Department of Psychology, University of Cambridge, Cambridge, United Kingdom; Hospital Universitari de Bellvitge, SPAIN

## Abstract

The increasingly widespread use of mobile phone applications (apps) as research tools and cost-effective means of vast data collection raises new methodological challenges. In recent years, it has become a common practice for scientists to design apps that run only on a single operating system, thereby excluding large numbers of users who use a different operating system. However, empirical evidence investigating any selection biases that might result thereof is scarce. Henceforth, we conducted two studies drawing from a large multi-national (Study 1; *N* = 1,081) and a German-speaking sample (Study 2; *N* = 2,438). As such Study 1 compared iOS and Android users across an array of key personality traits (i.e., well-being, self-esteem, willingness to take risks, optimism, pessimism, Dark Triad, and the Big Five). Focusing on Big Five personality traits in a broader scope, in addition to smartphone users, Study 2 also examined users of the main computer operating systems (i.e., Mac OS, Windows). In both studies, very few significant differences were found, all of which were of small or even tiny effect size mostly disappearing after sociodemographics had been controlled for. Taken together, minor differences in personality seem to exist, but they are of small to negligible effect size (ranging from OR = 0.919 to 1.344 (Study 1), η_p_^2^ = .005 to .036 (Study 2), respectively) and may reflect differences in sociodemographic composition, rather than operating system of smartphone users.

## Introduction

Following the advent and proliferation of smartphones, app-based research has spread across the scientific landscape, ranging from fields as diverse as physics [1], tourism [2,3] and geology [4,5] to medicine [6,7]. Of note, it falls on especially fertile grounds in psychology, booming throughout the discipline, from biopsychology [8] to neuroscience [9,10] and personality research [11, 12, 13]. The reasons for this momentum are manifold:

Smartphone technology enables researchers to collect an abundance of data (high volume), that arrives as a continuous stream in real time (i.e., high velocity) from various, multifaceted sources i.e., high variety [14, 15]. On top of experience sampling, i.e., the repeated, context-sensitive assessment of cognitive, affective, and behavioral measures across a certain period [16], modern smartphones may grant researchers access to global positioning system (GPS) based location data, communication logs, video and audio capture, motion sensing, and biosensors [14, 15, 17]. Linking these data to geographic information system data (e.g., climate and neighborhood characteristics) further allows scientists to paint an unprecedentedly fine-grained picture of people’s dynamic physical and social surroundings [18,19]. Leveraging this mechanism would facilitate to gain an in-depth understanding of the complex person × environment interactions that shape human behavior and might spur breakthroughs in personality and social psychology [19].

From a users’ perspective, science apps (i.e., mobile phone applications that serve scientific purposes as a research tool) are highly convenient as they do no longer require participants to be physically present in a lab [20] or, as in early Internet-based research, at a desktop or laptop computer. Increasingly often, both data collection and transmission happen automatically in the background, effectively reducing participant burden to a minimum [8,15].

Similarly, unlike their predecessors, personal digital assistants (PDAs), smartphones are already established as an integrated part of our lives [11,21]. Spending considerable amounts of time with smartphones has become a standout lifestyle feature [22], reflecting the contemporary philosophy of life in modern societies.

Given the remarkable growth rates of the smartphone market, the number of people carrying a smartphone is expected to skyrocket from 1 billion at the beginning of the decade [21,23,24], and 2 billion in 2016 [25] to over 5 billion by 2025 [17]. Against this backdrop, it is especially noteworthy, that smartphones have already begun to penetrate the emerging markets of developing countries and might soon become more common than computers [19]. In the absence of any available alternatives, in some regions smartphones may even constitute a monopoly-like structure, providing the only means to connect people to the Internet. In view of these tendencies, it appears encouraging, that data that has been submitted through mobile devices has been shown to be no less valid or reliable than data that was obtained from desktop users [26] or during laboratory experiments [23]. Marking the next leap towards a valid test of the universality of psychological theories [23], this trend thus has the capacity to pave the way for the inclusion of previously under-studied populations and ultimately, a wider coverage of cross-cultural research [20].

In a nutshell, smartphones are ubiquitous, fairly unobtrusive, remotely accessible, sensor-rich, and computationally powerful [17,22], thus setting the stage for the emerging field of *Psychoinformatics* at the crossroads of psychology and computer science [15,24,25]. Accordingly, smartphones may empower researchers to conduct large-scale longitudinal studies in real-world settings at low cost, featuring heterogeneous, global samples. Thanks to that, scientists may base future discoveries on an abundance of precise, and ecologically valid behavioral data above and beyond traditional self-reports [17,19, 27, 28]. Likewise, due to the remarkable computational powers, there are no apparent boundaries restricting the content of smartphone-based research and even highly complex cognitive tasks can be administered with ease [23]. Making use of these innovative possibilities would enable researchers to cross-validate, or challenge existing findings from lab settings [16,25] and extend the scientific body of knowledge beyond traditional Internet-based research that set out to achieve the same goals [29–31].

Thus, harnessing this untapped potential seems imperative. However, caution is warranted as some caveats (e.g., ethical and technical considerations with respect to data privacy and confidentiality, data transmission and storage solutions, security issues, app quality, and safety) prevail that need to be addressed for the sake of sound and adequate research practices [14,15,17,25, 27, 32]. In view of the constantly growing plethora of apps, guidance is needed to identify trustworthy, proper science apps. On a more methodological note, concerns have been voiced regarding potential technology-induced selection biases e.g., [33]. By definition, smartphone research is limited to the population of smartphone owners, creating coverage issue [34]. Yet, this might be less problematic, given the afore-mentioned rapidly expanding distribution of smartphones worldwide that will soon rise to a level of almost complete coverage.

Moreover, even though Lane and Manner [35] did find that smartphone ownership was predicted by extraversion, the authors have argued themselves that personality is, overall, a rather weak predictor of smartphone ownership. Hence, we tentatively conclude that it appears appropriate to assume that coverage issues, i.e. differences between smartphone owners and non-smartphone owners do not undermine the generalizability of app-based research to a worrying extent. Nevertheless, this does not rule out other systematic biases within the population of smartphone users.

Conducting a thorough analysis of the major smartphone operating systems (OS) in terms of their suitability as a research tool (i.e., Android, iOS, BlackBerry, Symbian, and Windows Mobile), Oliver [36] concluded that while every platform has its pros and cons, none of them is ideal or even generally superior to its competitors. As a solution, researchers could develop several native science apps in the respective programming languages or come up with web-based hybrid apps by means of cross-platform development tools (CPDTs). Alternatively, if social scientists do not see themselves fit for programming, interdisciplinary collaborations with trained computer scientists may prove effective [27].

However, while studies that accommodate both major systems (i.e., Android and iOS) do exist [37,38], they are much more of the exception rather than the general rule. In contrast, it is fairly common for psychological app-based studies to be run solely on either Android [39–42], iOS [43–46] or a different OS [13,47].

Perhaps puzzling at first, this pattern can be explained as follows: Programming multiple apps, one for each system is a tedious and time-consuming process that requires, above all, sufficient knowledge and skills in at least two programming languages, which makes it less desirable. In the meantime, CPDTs are still developing, often failing to live up to the performance of their native counterparts [48, 49]. To make things worse, Miller [17] has pointed out that there is currently only a small minority of psychologists, sufficiently tech-savvy and advanced in computer science to program apps efficiently by themselves. While it is hence understandable, that researchers shy away from coming up with science apps that accommodate both systems, it might jeopardize the data’s generalizability if user personality is related to smartphone operating systems as much as it is related to computer operating systems [33].

In other words, if iOS and Android users were to differ systematically regarding fundamental psychological characteristics, results of smartphone app studies would be inherently biased and per se compromised in their external validity. This would be a particularly harsh setback for the burgeoning field of personality research in *Psychoinformatics* [11,12,13], whose results would become questionable at best. As, to our knowledge, no study has examined this possibility so far, we aim to compare iOS and Android users along an array of personality traits.

To that end, we come up with two studies that complement each other.

More precisely, Study 1 employs a holistic personality assessment to screen for potential differences in various diverse traits across a large multi-national sample. Beyond the Big Five personality traits [50] at the core, it seeks to capture other facets of user personality that tap into different aspects and may therefore add incremental value and explanatory power. As such it draws from positive psychology by collecting data on well-being [42], global self-esteem [51] and optimism [52]. In juxtaposition, it also turns to more sinister traits, namely risk proneness [53], pessimism [52] and the Dark Triad (i.e., narcissism, psychopathy, and Machiavellianism; [54]).

Building on that, Study 2 aims to consolidate those findings and further extend the scope of our research to the computer realm, drawing from an even bigger, German-speaking sample and takes not only iOS and Android, but also Windows and Mac OS users into account (throughout the remainder of this article, Mac OS refers to the computer division of Apple, i.e. Mac operating systems that run on iMacs and MacBooks.). In recognition of the pre-eminent position of the Big Five taxonomy, as the predominant personality framework in mobile phone and Internet studies [12,55], and in the absence of notable effects for the other personality traits in Study 1, Study 2 is deliberately restricted to the Big Five [50]. This approach limits error due to multiple testing issues [12] and together with the enhanced statistical power, arising from the large sample, allows for even more rigorous testing. Study 2 improves further on Study 1 in assessing participants’ OS non-reactively, i.e., automatically upon accessing the questionnaire, thereby avoiding self-reports, which are prone to evoke biases. Given their overlapping, yet complementary design, we believe that if the results of Study 1 and Study 2 converge, one could claim with some confidence that indeed, personality differences between users of different operating systems do–or do not exist.

### Research questions and hypotheses

In marketing research and consumer psychology, brands are believed to have a personality, featuring a unique set of characteristics usually attributed to humans [56]. Henceforth, attitudes towards specific brands can be formed on the basis of these personality traits. Accordingly, these attitudes may serve the purpose of allowing consumers to express their self-concepts through the purchase, use and ownership of particular brands [57].

Reflecting its rather unique firm philosophy and marketing strategy, the Apple brand personality was built to convey qualities such as nonconformity, innovation, and creativity [58]. Unlike PC in the computer domain, or Samsung, SONY, and Nokia in the smartphone sector, Apple has successfully managed to become a lifestyle brand, echoing a modern, youthful philosophy of life that rests on the pillars of freedom, imagination, and simplicity at the heart of a seemingly truly humanistic, caring company. Lending empirical support to these observations, research has shown that whereas consumers describe Apple as exciting, SONY is rather seen as competent and sincere [59].

Moreover, the iPhone has become a status symbol for some people, inducing a feeling of belonging to a societal avant-garde in those who carry it [60]. Contrarily, consistent with its strategy to target the mass market, Samsung has cultivated a fairly different brand personality, emphasizing values such as ruggedness and functionality [60]. Summed up, on the one hand Apple stands for an outgoing, adventurous and lively brand personality, on the other hand it gives rise to an elitist self-definition of its customers, who may seek social approval and boost their self-esteem by being identified with Apple products. Meanwhile, Android brands (e.g., Samsung, SONY) appear to promote a more down-to-earth approach, grounded in a reliable, but significantly less fancy and glamorous product assortment. While we do not want to give in to mere speculations, drawing from the presented findings, we formulated the following hypotheses:

#### Hypothesis 1 (Study 1)

*On average*, *iOS users will score higher on global self-esteem than Android users*, reflecting the widespread belief that whereas the iPhone is a status symbol that carries prestige, fashionability, and exclusivity, all of which are suitable to make one feel valued and special, thus promoting enhanced self-esteem, Android smartphones fail to exert this same power.

#### Hypothesis 2 (Study 1, Study 2)

*On average*, *iOS users will show higher Extraversion than Android users*. Owing to Apple’s brand image as young, daring, outgoing and creative–an array of personality characteristics that seems to be rather closely linked to an extraverted personality, enhanced extraversion can be expected in accordance with the notion, that brand personality is supposed to mirror one’s own personality.

As neither the existing literature, nor common sense would allow similarly specific predictions, we refrained from formulating additional hypotheses for the other variables. Nonetheless, we believe that the inclusion of these constructs is conducive to the overall aim of the present research which is to detect any noteworthy personality differences as a function of users’ OS. Henceforth we tried to accomplish the most extensive coverage of user personality given existing constraints (e.g., questionnaire length) and adopted an exploratory approach in the search of potential differences. Likewise we investigated whether participants’ language (i.e., English or German) moderated the observed links, without holding any directional expectations.

Because there is very litte research on the topic of personality differences regarding used operating systems, we assumed a low effect size (*d* = 0.2 and η_p_^2^ = 0.01 according to [61]). A power analysis (α = 5%, power = 80%, two-tailed) recommends a minimal sample size of *N* = 788 for Study 1 and *N* = 1,096 for Study 2.

## Materials and methods–study 1

### Participants

The sample was comprised of 1,081 participants, 624 (58%) of whom reported to be female, while 449 (41%) reported to be male, and 8 (1%) who did not disclose their sex. Reported age ranged from 18 to 94 years (*M* = 24.5, *SD* = 8.1). Recruitment ensued online on various national and international platforms (e.g., Facebook, reddit), as well as on campus at the University of Konstanz, Germany, by word-of-mouth and custom-tailored advertisement of the study in introductory psychology lectures. Following this twofold strategy, the obtained sample comprised 507 participants (46.9%) from German-speaking countries (Germany: 44.8%, Switzerland: 1.2%, Austria: 0.9%) and 574 participants (53.1%) who were either from English-speaking countries or mastered English fluently (USA: 25.5%, Australia: 3.9%, UK: 2.5%, Canada: 2.3%).

Reported monthly budget ranged from less than 250€ to 5,001€ or more, with 76% of the sample disposing of 2,000€ or less per month, while 11% chose not to reveal their monthly budget. Regarding OS usage, 573 participants (53.0%) identified themselves as Android users, 444 participants (41.1%) indicated they use an iPhone. Meanwhile a small proportion indicated that they use either a Windows Phone (3.3%), a completely different operating system (1.1%), or no smartphone at all (1.3%). For parsimony’s sake only users of the two main OS (i.e., Android and iOS) were considered for further analysis resulting in a final sample size of 1,017.

Furthermore, the participant pool was mostly made up of college students (65.6%), active members of the workforce (31.7%), and high school students (8.8%), while others were unemployed (2.7%) or did not disclose their occupation (2.0%). (Please note that the accumulated percentages may exceed 100 percent, as participants could indicate multiple occupations, e.g. being a college student while working full-time.) The majority of the sample reported to be single (48.3%) or currently engaged in a romantic relationship (39.5%), while small fractions were married (7.3%), divorced (0.6%), or widowed (0.1%), or did not report their present marital status (2.1%).

### Materials

Questionnaire length in electronically distributed online surveys deserves special attention, as the same content may appear longer on Web sites, stretching across several pages, as opposed to traditional paper-and-pencil questionnaires [62], also see [63], for the one-item-one-screen design. Furthermore, dropout decisions are based on study attributes, such as survey length [64] or incompatibilities of technology used [65]. Similarly, previous research has shown that dropout risk rose by 40% from a 10-minute questionnaire to a 30-minute questionnaire [66]. Motivated by those findings, we deliberately decided to limit the online questionnaire to a restricted number of items that would take no longer than 15 minutes to complete, in order to decrease participant burden and, in turn, foster participation. Henceforth, aside from a small battery of demographic questions, we aimed to employ short, yet effective measures that are well-suited for group-level analysis [67,68] and possess satisfying psychometric properties [67].

In line with this rationale, we assessed Big Five personality traits with the Mini-IPIP [50], which contains 20 items and has repeatedly been shown to have acceptable reliability estimates [69,70].

Moreover, we chose to gauge global self-esteem by means of the Single-Item Self-Esteem Scale (SISE) that has been successfully translated into other languages before [71] and demonstrated to be of satisfactory validity [51]. Similarly, we employed a single-item measure of well-being, which has been a robust indicator in previous research with German-speaking samples [42].

Furthermore, we chose the Dirty Dozen [54] as a representative of negative personality attributes, consisting of 12 items, which have been shown to be an efficient, psychometrically acceptable measure of the Dark Triad [72].

Apart from that, we employed some short scales, which originate from the Leibniz Institute for the Social Sciences (GESIS) and have been validated on large, stratified samples, to measure the following constructs: risk proneness (1 item), [53], optimism and pessimism (2 items), [52]. In addition, we also assessed social desirability (6 items; 2 subscales), [73].

However, both subscales yielded unacceptably low reliability estimates (NQminus α = .564, PQplus α = .495) and were henceforth not considered for any further statistical analysis. Aside from the said GESIS measures (i.e., optimism, pessimism, risk proneness), which have been validated extensively on large, stratified German samples, it was ensured that the German versions of our instruments had been translated by professionals and repeatedly used in previous studies so that their appropriateness and precision could be taken for granted (see [74] for Mini-IPIP, see [42] for well-being). The only exceptions were the Dirty Dozen and the SISE. In the absence of established German versions, the scales were translated from the original English using the parallel-blind technique [75].

### Procedure

The survey was designed for optimization on regular computers and smartphones, using the SoSci Survey online tool (https://www.soscisurvey.de/). The questionnaire was administered online and available for a period of 3 months in English and German. As a general rule, participation was unpaid and voluntary, without further incentives, such as personalized feedback from the questionnaire.

However, psychology students, enrolled at the University of Konstanz, were offered course credit for participation. Beyond that, upon inclusion in the sample, participants automatically entered a lottery, raffling off Amazon gift card vouchers of a value of 100€ in total, unless they specifically requested otherwise.

### Ethics

The present study was conducted in accordance with the Ethical Guidelines of the German Psychological Society (DGPs) and the Ethical Guidelines of the Department of Psychology, University of Konstanz. Formal ethics approvals for this type of research (i.e., noninvasive, not affecting the physical or psychological integrity, the right for privacy or other personal rights of interest) are required neither by these guidelines nor by German laws.

All participants consented to the terms of the study, which were outlined in detail, preceding the actual questionnaire. As such, providing informed consent was made a prerequisite to proceed to the main part of the survey. Participants were explicitly told that they could revoke their consent and withdraw from the study at any time without any personal disadvantages arising from it. Furthermore, anonymity was ensured and no harmful procedures were applied. The same precautions and ethical standards were also upheld throughout Study 2.

### Statistical analysis

Following a twofold analysis procedure, we initially checked for potential differences in demographic variables, between self-reported iOS and Android users. Hereafter, we employed inferential statistics to account for possible distinctions with respect to the available personality measurements, beyond the influences of sociodemographic variables. Thereby we conducted both, confirmatory and exploratory analyses.

## Results–study 1

### Demographics

At first, we ran a series of χ^2^-tests to investigate the sample’s demographic composition. In this context, we did neither detect any significant differences for marital status (χ^2^ = 4.18, *df* = 4, *p* = .38), nor for participant’s sex (χ^2^ = 1.03, *df* = 1, *p* = .31). Similarly, a *t*-test failed to unveil any significant differences in reported age between iOS users (*M* = 24.23, *SD* = 8.10) and Android users (*M =* 24.40, *SD* = 7.63), *t* = -0.358, *p* = .72. However, significant differences although of very low effect size emerged in terms of the distribution of participants’ monthly budget, with iOS users tending to have access to somewhat larger financial resources (χ^2^ = 22.75, *df* = 9, *p* = .007; *r*_sp_ = .07).

### Personality traits

Given the multitude of variables and the risk of type I error that would have resulted from multiple testing, when conducting individual ANOVAs for every trait, we decided to run a binary logistic regression model instead, whereby smartphone OS (i.e., iOS vs. Android) was predicted by well-being, SISE, risk proneness, optimism, pessimism, the Dark Triad, the Big Five as well as sex, age and monthly budget. To that end, we employed a hierarchical analysis approach, featuring three stages and thus a step-wise increase in our model’s complexity.

First, we entered the sociodemographic variables (i.e., sex, age, and monthly budget) alone to predict users’ OS. Second, we entered both the sociodemographic variables and the personality traits (i.e., well-being, SISE, risk proneness, optimism, pessimism, Dark Triad, Big Five) to see whether this would lead to a significant improvement of the model’s fit to the data above and beyond the predictive power of sociodemographic factors. Third, in order to consider moderating effects that might arise from differences grounded in language or culture, we decided to rerun the full model (step 2) independently for the English-speaking and German-speaking subsamples. Please note, that separate ANOVAs (respectively ANCOVAs when controlling for sociodemographic variables) provide a more fine-grained picture and allow to tease out personality traits’ individual contributions. However, the results remain largely unchanged and the little effects that emerge mostly disappear when controlling for age, sex, and monthly budget. Additional ANOVA-based analyses are available in an online supplement (see S1 Table).

Overall, the data demonstrated that differences between iOS and Android users were largely absent. While Model 1 was significant and accounted for 1.6% of variance (Nagelkerke *R*^*2*^ = .016) entering the personality constructs in a second step significantly improved the predictive power of the model (step: χ^2^ = 23.700, *df* = 13, *p* = .034), with the proportion of explained variance rising to 5% (Nagelkerke *R*^*2*^ = .050) (see Table 1, column 1 and 2). Moreover, of all variables only two emerged as statistically significant predictors of user OS. Higher monthly budget predicted a higher likelihood of using iOS (*OR* = 0.922), whereas Openness to Experience was related to an increased probability of using Android (*OR* = 1.343). However, in both cases Odds Ratios gravitated towards 1.0, indicating a weak relationship and were far off common thresholds of a strong effect (e.g., 3.0 for positive associations, [75]).

**Table 1:** Binary logistic regression predicting smartphone OS (0 = iOS, 1 = Android).

	Model 1—Entire sample		Model 2—Entire Sample		Model 2a - English subsample		Model 2b - German subsample	
	*R*^*2*^ = .016; χ*^2^* = 10.596*		*R*^*2*^ = .050; χ*^2^* = 34.296*		*R*^*2*^ = .078; χ*^2^* = 26.852*		*R*^*2*^ = .053; χ*^2^* = 18.168	
	*N* = 905		*N* = 905		*N* = 446		*N* = 459	
	*b*	*OR* [95% *CI*]	*b*	*OR* [95% *CI*]	*b*	*OR* [95% *CI*]	*b*	*OR* [95% *CI*]
Sex	0.063	1.066 [0.812, 1.398]	0.022	1.022 [0.746, 1.401]	0.305	1.356 [0.884, 2.082]	-0.256	0.774 [0.472, 1.269]
Age	0.014	1.015 [0.996, 1.034]	0.008	1.088 [0.988, 1.027]	0.014	1.102 [0.989, 1.041]	-0.007	0.993 [0.961, 1.026]
Monthly budget	-0.085*	**0.919 [0.872, 0.969]**	-0.081*	**0.922 [0.873, 0.974]**	-0.069*	**0.934 [0.877, 0.993]**	0.001	1.001 [0.858, 1.167]
Well-being	–	–	0.000	1.000 [0.990, 1.011]	-0.001	0.999 [0.984, 1.013]	0.004	1.004 [0.988, 1.021]
Self-esteem	–	–	-0.038	0.963 [0.782, 1.170]	-0.085	0.918 [0.698, 1.207]	0.033	1.034 [0.774, 1.381]
Willingness to take risks	–	–	-0.093	0.912 [0.816, 1.019]	-0.074	0.928 [0.779, 1.106]	-0.068	0.934 [0.798, 1.093]
Optimism	–	–	-0.084	0.919 [0.784, 1.077]	-0.021	0.980 [0.793, 1.209]	-0.069	0.934 [0.720, 1.211]
Pessimism	–	–	0.170	1.017 [0.876, 1.180]	-0.086	0.918 [0.755, 1.116]	0.221	1.248 [0.973, 1.600]
Dark Triad: Machiavellianism	–	–	-0.056	0.945 [0.850, 1.052]	-0.017	0.984 [0.846, 1.114]	-0.800	0.923 [0.789, 1.080]
Dark Triad: Psychopathy	–	–	0.066	1.068 [0.945, 1.207]	0.032	1.032 [0.866, 1.230]	0.054	1.055 [0.873, 1.276]
Dark Triad: Narcissism	–	–	-0.049	0.952 [0.866, 1.047]	-0.032	0.969 [0.840, 1.118]	-0.077	0.926 [0.811, 1.057]
Big Five: Extraversion	–	–	-0.054	0.947 [0.788, 1.138]	-0.236	0.790 [0.612, 1.019]	0.083	1.087 [0.818, 1.444]
Big Five: Agreeableness	–	–	0.008	1.008 [0.803, 1.264]	-0.010	0.990 [0.718, 1.364]	-0.187	0.829 [0.575, 1.195]
Big Five: Conscientiousness	–	–	0.039	1.040 [0.866, 1.249]	-0.048	0.953 [0.730, 1.244]	0.086	1.090 [0.833, 1.425]
Big Five: Neuroticism	–	–	-0.114	0.892 [0.724, 1.099]	0.072	1.075 [0.793, 1.457]	-0.288	0.750 [0.551, 1.019]
Big Five: Openness to Experience	–	–	0.295*	**1.343 [1.087, 1.659]**	0.295*	**1.344 [1.010, 1.787]**	0.305	1.357 [0.976, 1.885]

*Note*. *R*^*2*^ = Nagelkerke; bold values indicate significance

* *p* < .05.

After the language-based split of the sample was performed to unpack potential cultural differences, almost identical patterns were observed in the English subsample (see Model 2a, Table 1, column 3). In contrast, the model for the German sample could not reliably predict smartphone OS above chance level and dropped below the threshold of statistical significance. Accordingly, no single predictor reached statistical significance. Of note, however, both Neuroticism (*b* = -0.28, *p* = .066, *OR* = 0.750, 95% CI: 0.551, 1.019) and Openness to Experience (*b* = 0.305, *p* = .069, *OR* = 1.357, 95% CI: 0.976, 1.885) approached statistical significance, with the latter mirroring the effect that was observed in the other models (see Model 2b, Table 1, column 4).

In the absence of any significant differences between iOS- and Android users in self-esteem (H1) or Extraversion (H2), none of our hypotheses received empirical support, although Extraversion did approach statistical significance (*b* = -0.236, *p* = .081, *OR* = 0.790, 95% CI: 0.612, 1.019) in the English subsample, showing a trend in the hypothesized direction.

In sum, our data suggest that iOS- and Android users show only minimal differences regarding psychological concepts. If anything, Android users tend to be a little more open, while iOS users may be slightly wealthier. Yet, all effect sizes were small to tiny. While Table 2 provides a summary of the measures’ descriptive statistics, detailed results of the logistic regression model are displayed in Table 1.

**Table 2:** Descriptives of study 1 variables separated for operating systems (Android, iOS).

	Cronbach α	Android	iOS
		*M* (*SD*)	*M* (*SD*)
Well-Being	^1)^	74.92 (17.99)	76.49 (17.58)
Single-Item Self Esteem	^1)^	3.44 (0.94)	3.52 (0.93)
Willingness to take risks	^1)^	4.23 (1.37)	4.47 (1.39)
Optimism	^1)^	4.85 (1.42)	5.05 (1.42)
Pessimism	^1)^	3.39 (1.43)	3.28 (1.47)
Dark Triad: Machiavellianism	.81	3.13 (1.65)	3.45 (1.73)
Dark Triad: Psychopathy	.66	3.26 (1.49)	3.24 (1.58)
Dark Triad: Narcissism	.78	4.36 (1.70)	4.62 (1.75)
Big Five: Extraversion	.81	3.08 (0.92)	3.19 (0.92)
Big Five: Agreeableness	.81	4.08 (0.78)	4.05 (0.76)
Big Five: Conscientiousness	.67	3.52 (0.80)	3.50 (0.81)
Big Five: Neuroticism	.72	2.85 (0.87)	2.89 (0.81)
Big Five: Openness to Experience	.64	4.00 (0.66) *N* = 573	3.88 (0.69) *N* = 444

Note.

^1)^ one-item measure

## Materials and methods–study 2

### Participants

Our second sample differed from the first sample insofar, as it was larger and more homogeneous with respect to its cultural composition. Several research assistants sent the link to the online questionnaire to their friends, relatives, and acquaintances by using various online channels (e.g., Facebook, Email). This snowball sampling procedure led to a community-based convenience sample of German-speaking participants, which was effectively reduced to 2,036 German-speaking participants due to the following reasons:

First, we excluded data sets from 26 participants who had exhibited suspicious responses that raised doubts about their seriousness in answering the questionnaire (always giving the same highly implausible answer to the delinquency questions, e.g., ‘99’). Second, as the study did specifically aim to compare Android-, iOS-, Windows, and Mac OS users, 145 participants relying on other operating systems were not considered for further analysis. In the final sample, 1,345 participants (66.1%) reported to be female and 685 participants (33.6%) reported to be male, 6 participants chose not to disclose their sex (0.3%). Age ranged from 18 to 78 years (*M* = 25.5, *SD* = 11.64). Furthermore, the vast majority (79.3%) had at least graduated from high school.

### Materials

In line with the approach adopted in Study 1 we chose to design the online questionnaire in a fashion that would allow participants to complete it in no more than 15 minutes for the sake of enhanced retention rates and increased data quality. Paralleling the procedure of Study 1, we relied on the Mini-IPIP [50] to measure Big Five personality traits. Due to a technical failure in the online questionnaire, one item of the Conscientiousness subscale was asked twice and one item was not asked. Therefore, the mean score of the Conscientiousness subscale is only based on three instead of four items. On top of this we also used a short battery of questions revolving around delinquency, which were part of a different research project and are henceforth not touched upon in the scope of this article.

### Procedure

The questionnaire was developed in accordance with standards for optimal depiction on both, regular computers and smartphones, using the SoSci Survey online tool (https://www.soscisurvey.de). As such, it was exclusively accessible online for a period of two months, and was in German only. In the absence of any financial or otherwise incentives, participation was per se unpaid and voluntary. The same ethical precautions and procedures were applied as in Study 1.

## Results–study 2

We followed a similar analysis procedure as outlined in Study 1. However, unlike Study 1, Study 2 included smartphone and computer users alike, resulting in four groups that were compared with each other (i.e., Mac OS, Windows, iOS, Android). As this design would have required running a multinomial regression analysis, with three different models (changing the reference group to determine pairwise group differences) per column, we chose to compute ANOVAs and ANCOVAs instead, which were deemed more parsimonious and easily comprehensible in the given context. Moreover, compared to Study 1, adopting this method bore a considerably smaller risk of suffering from multiple testing issues due to the reduced set of variables.

### Demographics

First, we carried out χ^2^-tests to account for the distribution of primary demographic attributes across the OS groups. Results yielded significant results for sex (χ^2^ = 27.44, *df* = 3, *p* < .001, φ = .116) and educational level (χ^2^ = 63.54, *df* = 18, *p* < .001, φ = .177), reflecting a deviation from a balanced distribution between the respective OS groups. Distribution of participants’ sex was rather balanced among Android users (standardized residuals -0.2 for women and 0.3 for men), slightly more male-dominated among Windows users (standardized residuals -0.7 and 1.0, respectively), clearly male-dominated among Mac OS users (standardized residuals -2.0 and 2.9, respectively), and clearly female-dominated among iOS users (standardized residuals 2.1 and -3.0, respectively). With respect to educational level, computer users were more likely to have graduated from university than users using smartphones (standardized residuals: Mac OS, college degree: 2.1, Windows, college degree: 4.1, iOS, college degree: -1.8, Android, college degree: -3.1).

In line with this finding, an ANOVA yielded significant age differences (*F* = 51.88, *df* = 3, *p* < .001, η_p_^2^ = .071), with Scheffé post-hoc tests indicating that Windows users (*M* = 30.1, *SD* = 15.4) were on average significantly older than Mac OS users (*M* = 27.2, *SD* = 12.2) who were, in turn, on average older than Android users (*M* = 23.3, *SD* = 8.8), and iOS users (*M* = 23.0, *SD* = 8.5). Given this pattern we would like to suggest that the differences in educational level might actually stem from age differences, suggesting that smartphone users in our sample may not yet have graduated from college despite proactively pursuing a higher education.

### Personality traits

In order to give a general overview, Table 3 exhibits descriptive parameters. The inferential analysis was carried out in two stages. At first, we ran ANOVAs on Big Five personality traits (Table 4, 1st and 2nd column). Thereafter, we conducted an ANCOVA to control for potential moderating effects of age, participant sex, and educational level (Table 4, 3rd and 4th column). Finally, we computed pairwise mean-differences (Bonferroni corrected) to pinpoint the concrete nature and direction of effects between the existing subgroups (Table 4, 5th column).

**Table 3:** Descriptives of study 2 variables separated for operating systems (Android, iOS, Mac OS, and Windows).

	Cronbach α	Android	iOS	Mac OS	Windows
		*M* (*SD*)	*M* (*SD*)	*M* (*SD*)	*M* (*SD*)
Big Five: Extraversion	.76	3.43 (0.80)	3.65 (0.76)	3.57 (0.76)	3.24 (0.86)
Big Five: Agreeableness	.77	4.29 (0.69)	4.28 (0.66)	4.20 (0.76)	4.23 (0.72)
Big Five: Conscientiousness	.56	3.63 (0.70)	3.61 (0.71)	3.63 (0.68)	3.62 (0.72)
Big Five: Neuroticism	.73	2.82 (0.86)	2.88 (0.86)	2.67(0.91)	2.76 (0.85)
Big Five: Openness to Experience	.63	3.73 (0.71)	3.72 (0.70)	3.95 (0.70)	3.83 (0.72)
		*N* = 765	*N* = 535	*N* = 160	*N* = 576

**Table 4:** Differences between operating systems (Android, iOS, Mac OS, and Windows; Study 2).

	All data			Controlled for age, sex, and education		
	*F*	η_p_^2^	Post-hoc Test (Scheffé)	*F*	η_p_^2^	Pairwise comparisons of main effect *operating system* (Bonferroni corrected)
Big Five: Extraversion	25.52***	.036	MAC > W, iOS > W, A > W, iOS > A	4.58**	.007	iOS > W
Big Five: Agreeableness	1.27	.002	One group	1.50	.002	None
Big Five: Conscientiousness	0.12	< .001	One group	0.53	.001	None
Big Five: Neuroticism	3.55*	.005	iOS > MAC	0.99	.002	None
Big Five: Openness to Experience	7.07***	.010	MAC > A, MAC > iOS	0.68	.001	None

*Note*. W = Windows, MAC = Mac OS, A = Android.

* *p* < .05

** *p* < .01

*** *p* < .001.

In resemblance with the patterns from Study 1, no significant differences between OS groups were detected, except for the Big Five personality traits Extraversion (η_p_^2^ = .036) and Openness to Experience (η_p_^2^ = .010), both of which were of rather small effect size (small: η_p_^2^ = .01, medium: η_p_^2^ = .06, large: η_p_^2^ = .14, 76). Of note, the difference for Neuroticism also reached statistical significance, although with a tiny effect size of η_p_^2^ = .005. However, the observed differences for Neuroticism and Openness to Experience vanished once age, sex, and educational level were controlled for (Table 4). Solely the reported differences for Extraversion (η_p_^2^ = .007) prevailed, even after accounting for the afore-mentioned moderators—although with a sharp drop in effect size.

Windows users displayed the lowest values on Extraversion, differing significantly from iOS- and Android users, both of which exhibited more extraversion. With respect to Hypothesis 2, this outcome fails to elicit significant differences between iOS- and Android users, and, like the Study 1 does not lend support to the hypothesis. However, it should be noted that the hypothesized differences were found before controlling for sociodemographic variables. All analyses are summarized in Table 4.

In a nutshell, very much alike Study 1, we demonstrated that in spite of a few significant differences between the users of the most prominent operating systems in key psychological concepts, namely Big Five personality traits, those differences are of small to tiny effect size. In our present studies, controlling statistically for age, sex, and educational level led to an almost complete disappearance of said effects.

## General discussion

Today, the rise of smartphones is already transforming our lives and will most likely continue to do so in the next years to come, as mobile technology becomes more and more ubiquitous all around the world. The new technology now impacts on various domains of our lives, yielding manifold consequences that echo throughout society. In the future this trend may be further amplified as everyday objects, e.g., fridges and cars will harbor remarkable computational powers and constant Internet connectivity, giving rise to the *Internet of Things (IoT)* [15, 25].

While it is widely anticipated that social scientific research will benefit from leveraging the enormous potential of these technologies, a number of methodological, technical, ethical, and practical hurdles peculiar to smartphone-based research prevail, which need to be dealt with, first (e.g., data privacy and confidentiality, data transmission and storage solutions, security issues, app quality, and safety; [14,15,17,27,32]).

Raising and addressing another issue linked to science apps, the present studies aimed to provide a clue as to whether researchers would need to accommodate both predominant smartphone operating systems (i.e., iOS and Android), in order not to jeopardize the generalizability of their findings.

For Study 1 a step-wise analysis procedure did yield a significant impact of Openness to Experience besides differences in sociodemographic variables. At first glance, this might pose a threat to the generalizability mentioned above. However, it is important to note, that all observed effects were of small or even tiny effect size in accordance with common classifications (e.g., [76]). Likewise, both our hypotheses, assuming differences in self-esteem and Extraversion, respectively, could not be confirmed and were henceforth rejected.

Bearing this in mind, it appears legitimate to assume that in spite of minor differences between iOS and Android users, none of the found differences are sufficiently strong to be of actual practical relevance. However, this impression may be misleading. On the contrary, we would like to stress that whereas it is relatively easy to statistically eliminate the influence of sociodemographic variables, it is by far less so when it comes to gathering actual samples via certain technologies. Replicating the classic study by Buchanan and Reips [33] the present results hint that in the given context, sociodemographic factors are a force to be reckoned with that exerts a sizable impact on the studied effects. This is reflected in the fact, that the only other significant predictor of smartphone OS, aside from Openness to Experience in Study 1 was monthly budget. In a similar vein, the observation, that most effects in Study 2 vanished after sociodemographic variables were controlled for, attests to the same possibility. Unless being accounted for by matched samples, by nature, the distribution of such sociodemographic variables may vary profoundly between operating systems. In conclusion, to avoid undue biases threatening the data’s validity, great care should be taken in terms of sample composition in science app studies, especially when recruiting ad-hoc samples.

### Strengths and limitations

In spite of our efforts to conduct the present research in the most beneficial and effective way, some drawbacks persisted nonetheless, which we intend to address in the following section. To start with, both studies used ad-hoc samples with very little recruitment restrictions. Although these community-based samples are more diverse in sample characteristics than common student samples and do henceforth generate a higher usability of the resulting data [77], some disadvantages need to be considered.

Notably, as a direct consequence, arising from our recruitment strategy, we faced a skewed sex distribution in the sample of Study 2, with roughly two thirds of the sample being women. This might sound worrying, because Big Five personality traits have been shown to vary as a function of sex, especially in well-developed wealthy and egalitarian societies just like Germany [78]. Both samples featured a rather wide range in terms of age, which is of interest as Big Five personality traits have also been reported to change dynamically across the lifespan [79]. This being said, one might turn this heterogeneity into an asset, as it reflects the actual age composition of the target population better than traditional psychological studies that are notoriously prone to draw from college student samples only [30]. As age was not even close to being a significant predictor of smartphone OS in Study 1, we are confident that the age distribution was fairly comparable between iOS and Android users and did not impair the results’ validity. Nonetheless, in keeping with the findings above on the link between sociodemographic variables and Big Five personality traits, we controlled statistically for sex, age, and educational level in Study 2. Of note, this had a strong influence on the obtained results that merits further attention.

From a methodological point of view, Study 2 may receive the critique that most people tend to own and use both, a smartphone and a computer system. Consequently their placement in the respective compared groups could be perceived as reflecting an arbitrary snapshot rather than a clear-cut, permanent membership in one particular user group (i.e., continuously favoring the usage of one electronic device over the other). Taking on this potential caveat, we analyzed switching patterns between smartphones and computers, drawing from a sizable longitudinal sample (*n* = 204) with an average of 48 data points per person, accumulating to a total 9,745 data points that has been collected in the frame of a different research project [80]. Consistent with our claim, results indicated that 92% of all participants kept using the same device in at least 80% of all data collection waves. Against this backdrop, it appears reasonable that a pronounced preference for a single electronic device exists in most people which allows to sort participants into the user groups that we employed throughout Study 2.

Furthermore, although we have mounted our best efforts to ascertain a holistic and balanced assessment of user personality, with a strong emphasis on the Big Five taxonomy, acknowledging its role as a key concept in smartphone-based personality research [11,12], we cannot rule out the possibility that we have failed to detect significant differences across users of different OS along unmeasured personality dimensions. While we tried to minimize this danger by assessing a host of vastly different characteristics to cover as much of users’ personality as possible, some traits may have fallen through the cracks, such as Gray’s reward sensitivity or, similarly, social desirability, which we did measure but could not analyze due to a lack of reliability. Faced with a length-breadth tradeoff, when designing our questionnaire, we chose to pursue a holistic, yet parsimonious approach to maintain participant motivation, reduce fatigue, boredom and dropout and yield high-quality data [64,66,67]. However, future research should expand on our findings and consider other personality traits.

While it is very clear, that our research leaves some room for improvement, it benefits from an array of assets that deserve to be mentioned. To start with, we would like to stress that thanks to its design, Study 2 can be interpreted as an in-built replication of Study 1, although with a somewhat narrower focus, concentrating on Big Five personality traits in a German-speaking sample. Beyond that, it makes two valuable contributions in extension of Study 1. Notably, we assessed OS, the grouping variable in question automatically, unlike Study 1 where we relied on self-reports. Moreover, it widens the horizon of the study, by taking desktop computer OS into account as well.

### Conclusion

Due to the novelty of smartphones in general and science apps in particular, a refined research philosophy as well as best practices to accommodate their use as data collection tools are currently still lacking. In recognition of the arising challenges, the present investigation represents an attempt to mark another step towards a robust, unified methodology for smartphone- and computer-based social science studies. Such studies provide an easy, yet cost-effective way of collecting vast amounts of ecologically valid data from diverse, geographically widely scattered samples. Events can be recorded in real time, as they occur.

Still, special care has to be taken, when employing smartphones and science apps, as an inadequate manner of using them for research purposes, may both, undermine data quality and compromise ethical standards. Against this backdrop, we aimed to shed new light on a potentially harmful selection bias that emerges following the widespread use of science apps that are compatible with one OS only. We argued, that if iOS and Android users were to differ significantly in personality, as marketing research and consumer psychology hint, the scientific community would need to introduce hybrid apps, or independently designed identical native apps for both systems, as a gold standard for app-research, for external validity’s sake. Thankfully for less tech-savvy scholars, according to our findings, this effort is not to be considered a necessity, in spite of potentially distorting differences in sociodemographic composition that researchers should be aware of. More to the point, minor differences in personality do exist, but they are of negligible effect size.

## Supporting information

S1 Table Pairwise Comparisons of personality traits between iOS and Android users (Study 1). *Note*. Bold values indicate significance (*p* < .05). small: η_p_^2^ = .010, medium: η_p_^2^ = .060, large: η_p_^2^ = .140; Cohen (1988). I…iOS, A…Android.
(PDF)
