# Supplementary material for: Users of the main smartphone operating systems (iOS, Android) differ only little in personality
Source: PLoS One. 2017 May 3;12(5):e0176921. doi: 10.1371/journal.pone.0176921 (PMC5415193; doi:10.1371/journal.pone.0176921)
Supplement: S1 Table — Note. Bold values indicate significance (p < .05). small: ηp2 = .010, medium: ηp2 = .060, large: ηp2 = .140; Cohen (1988). I…iOS, A…Android. (PDF) [file pone.0176921.s001.pdf]

Supporting Information:

Users of the main smartphone operating systems (iOS, Android) differ only little in personality

Friedrich Götz[1,2]

Stefan Stieger[1]

Ulf-Dietrich Reips[1]

[1] Department of Psychology, University of Konstanz, Konstanz, Germany

[2] Department of Psychology, University of Cambridge, Cambridge, United Kingdom

Correspondence to: Friedrich Götz, Department of Psychology, University of Cambridge, Downing Street, Cambridge CB2 3EB, United Kingdom. E-mail: fmg33@cam.ac.uk

A. Pairwise Comparisons of Personality Traits between iOS and Android Users

S1 Table. *Differences between iOS and Android (Study 1)*

	All data		German subsample		English subsample		German subsample		English subsample	
	N = 1,017		*N* = 483		*N* = 534		controlled for age, sex and monthly budget			
	F	η_p^2	*F*	η_p^2	*F*	η_p^2	*F*	η_p^2	*F*	η_p^2
Well-Being	$1.796^{I>A}$	.002	$0.134^{A>I}$	<.001	**4.315**$^{I>A}$	.009	$0.353^{A>I}$	.001	$2.475^{I>A}$	.007
Single-Item Self Esteem	$1.712^{I>A}$	.002	$0.086^{A>I}$	<.001	**4.187**$^{I>A}$	.008	$0.414^{A>I}$	.001	$2.839^{I>A}$	.007
Willingness to take risks	**7.269**$^{I>A}$	.008	$0.617^{I>A}$	.001	**3,937**$^{I>A}$	.008	$0.385^{I>A}$	.001	$3.027^{I>A}$	.008
Optimism	**4.660**$^{I>A}$	.005	$0.832^{I>A}$	.002	**4.640**$^{I>A}$	.010	$0.508^{I>A}$	.001	$0.957^{I>A}$	.003
Pessimism	$1.223^{A>I}$	.001	$1.526^{A>I}$	.003	$0.793^{A>I}$	.002	$0.804^{A>I}$	.002	$0.541^{A>I}$	.001
Dark Triad: Machiavellianism	**3.952**$^{I>A}$	.004	$2.960^{I>A}$	.006	$0.340^{I>A}$	.001	$3.419^{I>A}$	.008	$0.705^{I>A}$	.001
Dark Triad: Psychopathy	$0.045^{I>A}$	<.001	$0.125^{A>I}$	<.001	$0.156^{A>I}$	<.001	$0.279^{A>I}$	.001	$0.467^{A>I}$	.001
Dark Triad: Narcissism	**5.426**$^{I>A}$	.006	$3.732^{I>A}$	.008	$1920^{I>A}$	.004	$3.306^{I>A}$	.007	$0.389^{I>A}$	.001
Big Five: Extraversion	$3.669^{I>A}$	.004	$0.036^{I>A}$	<.001	**10.283**$^{I>A}$	.021	$0.020^{I>A}$	<.001	**4.888**$^{I>A}$	.013
Big Five: Agreeableness	$0.279^{A>I}$	<.001	$0.619^{I>A}$	.001	$0.120^{I>A}$	<.001	$0.714^{I>A}$	.002	$0.366^{I>A}$	.001
Big Five: Conscientiousness	$0.121^{A>I}$	<.001	$0.568^{A>I}$	.001	$1.185^{I>A}$	.002	$1.492^{A>I}$	.003	$1.062^{I>A}$	.003
Big Five: Neuroticism	$0.463^{I>A}$	<.001	$1.812^{I>A}$	.004	$0.532^{A>I}$	.001	$2.701^{I>A}$	.006	$2.244^{A>I}$	.006
Big Five: Openness to Experience	**8.383**$^{A>I}$	.009	$1.544^{A>I}$	.003	**6.760**$^{A>I}$	.014	$3.190^{A>I}$	.007	**3.898**$^{A>I}$	.010

Note. Bold values indicate significance ($p < .05$). small: $\eta_p^2 = .010$, medium: $\eta_p^2 = .060$, large: $\eta_p^2 = .140$; Cohen (1988). I…iOS, A…Android

In order to elucidate the contribution and contingencies of every predictor independently, we ran a series of ANOVAs (S1 Table, 1st, 2nd and 3rd column) and ANCOVAs (S1 Table, 4th and 5th column) analogous to the procedure of the binary logistic regression reported in the manuscript. As such our analysis featured three stages and thus a stepwise increase in the model's complexity:

1. ANOVAs across entire sample
2. Separate ANOVAs for English and German subsamples
3. ANCOVAs across English and German subsamples, controlling for age, sex, and monthly income.

Consistent with the reported findings in the article, in spite of a number of significant results, all of them were of small effect size at most (small: η_p^2 = .01, medium: η_p^2 = .06, large: η_p^2 = .14) [76]. Notably, for the undivided sample, significant differences with iOS users scoring higher occurred for willingness to take risks (η_p^2 = .008), optimism (η_p^2 = .005), Machiavellianism (η_p^2 = .004), narcissism (η_p^2 = .006), and with Android users scoring higher on Openness to Experience (η_p^2 = .009), see S1 Table, column 1.

Analyses within the English-speaking subsample revealed significant differences for well-being (η_p^2 = .009), self-esteem (η_p^2 = .008) willingness to take risks (η_p^2 = .008), optimism (η_p^2 = .010), Openness to Experience (η_p^2 = .017) and Extraversion (η_p^2 = .020). Whereas the former reflects higher scores for Android users, the latter indicates a higher degree of Extraversion among iOS users, consistent with Hypotheses 1 and 2 (S1 Table, 2nd column). In the German-speaking subsample none of the comparisons reached statistical significance (S1 Table, 3rd column).

In line with the findings from the logistic regression analysis, where monthly budget emerged as only significant predictor of users' OS, alongside Openness to Experience, controlling for age, monthly budget and sex led to the disappearance of almost all effects (see S1 Table, 4th and 5th column). Among German-speaking participants, no significant differences persisted. Turning to the English-speaking subsample, lending Hypothesis 2, we found that iOS users had higher scores on Extraversion (η_p^2 = .013), while the predicted effect for self-esteem dropped below the threshold of statistical significance, thus failing to support Hypothesis 1. Mirroring the outcomes of the logistic

regression, the only other significant predictor was Openness to Experience (η_p^2 = .010), which was higher among Android users than iOS users.

Summed up, in close resemblance to our findings from the logistic regression, although we found significant differences between iOS and Android users regarding several psychological concepts, all effect sizes were small to tiny and mostly disappeared when controlling for sociodemographic variables.
